# Harmful and beneficial aspects of *Parthenium hysterophorus*: an update

**DOI:** 10.1007/s13205-011-0007-7

**Published:** 2011-04-27

**Authors:** Seema Patel

**Affiliations:** Department of Biotechnology, Lovely Professional University, Jalandhar, 144402 Punjab India

**Keywords:** *Parthenium hysterophorus*, Sesquiterpene lactone, Dermatitis, Biocontrol, Green manure, Bioremediation

## Abstract

*Parthenium hysterophorus* is a noxious weed in America, Asia, Africa and Australia. This weed is considered to be a cause of allergic respiratory problems, contact dermatitis, mutagenicity in human and livestock. Crop production is drastically reduced owing to its allelopathy. Also aggressive dominance of this weed threatens biodiversity. Eradication of *P. hysterophorus* by burning, chemical herbicides, eucalyptus oil and biological control by leaf-feeding beetle, stem-galling moth, stem-boring weevil and fungi have been carried out with variable degrees of success. Recently many innovative uses of this hitherto notorious plant have been discovered. *Parthenium hysterophorus* confers many health benefits, viz remedy for skin inflammation, rheumatic pain, diarrhoea, urinary tract infections, dysentery, malaria and neuralgia. Its prospect as nano-medicine is being carried out with some preliminary success so far. Removal of heavy metals and dye from the environment, eradication of aquatic weeds, use as substrate for commercial enzyme production, additives in cattle manure for biogas production, as biopesticide, as green manure and compost are to name a few of some other potentials. The active compounds responsible for hazardous properties have been summarized. The aim of this review article is to explore the problem *P. hysterophorus* poses as a weed, the effective control measures that can be implemented as well as to unravel the latent beneficial prospects of this weed.

## Introduction

**Fig. 1:**
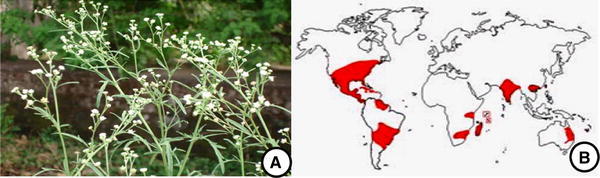
**a***P. hysterophorus* weed; **b** geographical distribution of *P. hysterophorus*

*Parthenium hysterophorus* is an aggressive ubiquitous annual herbaceous weed with no economic importance unravelled till now. This erect, ephemeral herb known for its vigorous growth and high fecundity especially in warmer climates is a native of north-east Mexico and is endemic in America. It is commonly known as ‘altamisa’, carrot grass, bitter weed, star weed, white top, wild feverfew, the “Scourge of India” and congress grass (Fig. 1a). *Parthenium hysterophorus* is a prolific weed belonging to Asteraceae family, producing thousands of small white capitula each yielding five seeds on reaching maturity. Within the past century it has found its way to Africa, Australia, Asia and Pacific Islands (Fig. 1b) and has now become one of the world’s seven most devastating and hazardous weeds. This noxious weed is often spotted on abandoned lands, developing residential colonies around the towns, railway tracks, roads, drainage and irrigation canals, etc. This weed grows luxuriantly in established gardens, plantations and vegetable crops. Due to its high fecundity a single plant can produce 10,000 to 15,000 viable seeds and these seeds can disperse and germinate to cover large areas.

This alien weed is believed to have been introduced into India as contaminants in PL 480 wheat (Public Law 480 passed in 1954 to give food grains to developing countries for eliminating starvation and malnutrition) imported from the USA in the 1950s. Presently, this invasive weed is widely prevalent in India (Singh et al. 2008). Approximately two million hectares of land in India have been infested with this herbaceous menace (Dwivedi et al. 2009).

Looking at the multitude of harms caused by *P*. *hysterophorus*, its management is necessary to prevent future problems. Since *P. hysterophorus* grows luxuriantly in many parts of the world, it is important to explore its beneficial uses if any. The purpose of this review article is to summarize the published papers in this area and highlight the menacing roles of the weed.

## Chemical analysis of *P. hysterophorus*

**Fig. 2:**
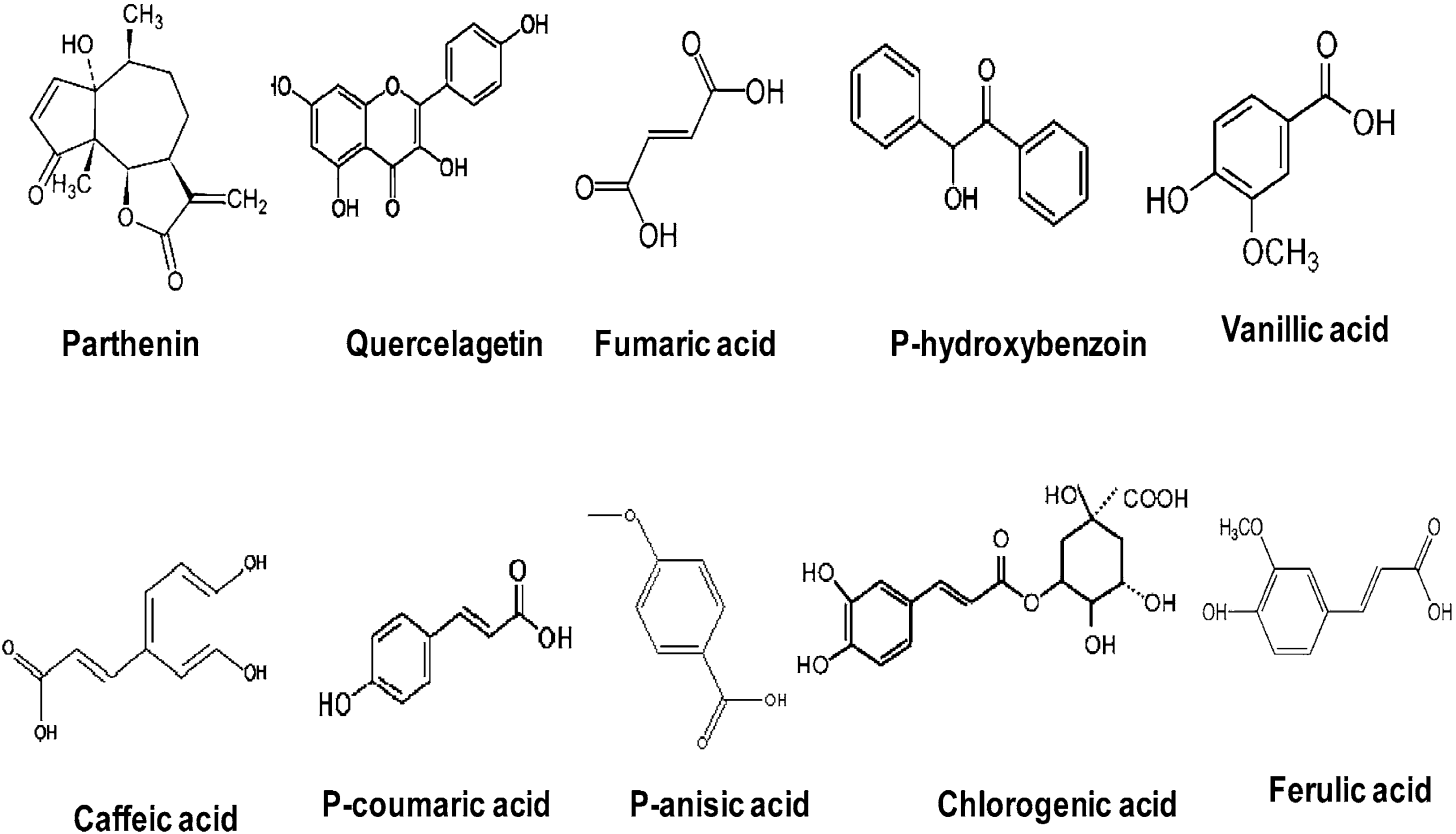
Sesquiterpene lactones from *P. hysterophorus*

Isolation and structural elucidation of the active principles of *P. hysterophorus* is required to determine their chemical properties. Chemical analysis of *P. hysterophorus* has indicated that all its parts including trichomes and pollen contain toxins called sesquiterpene lactones (SQL). Maishi et al. (1998) reported that *P. hysterophorus* contains a bitter glycoside parthenin, a major sesquiterpene lactone. Other phytotoxic compounds or allelochemicals are hysterin, ambrosin, flavonoids such as quercelagetin 3,7-dimethylether, 6-hydroxyl kaempferol 3-0 arabinoglucoside, fumaric acid. P-hydroxy benzoin and vanillic acid, caffeic acid, p courmaric, anisic acid, p-anisic acid, chlorogenic acid, ferulic acid, sitosterol and some unidentified alcohols (Fig. 2). Parthenin, hymenin and ambrosin are found to be the culprits behind the menacing role of this weed in provoking health hazards (Lata et al. 2008). *Parthenium hysterophorus* from different geographical regions exhibited parthenin, hymenin, coronopilin, dihydroisoparthenin, hysterin, hysterophorin and tetraneurin A as the principal constituents of their sesquiterpene lactones (De La Fuente et al. 1997). Gupta et al. (1996) identified a novel hydroxyproline-rich glycoprotein as the major allergen in *P. hysterophorus* pollen. Das et al. (2007) examined the flowers of *P. hysterophorus* and isolated four acetylated pseudoguaianolides along with several known constituents. A novel sesquiterpenoid, charminarone, the first seco-pseudoguaianolide, has been isolated along with several known compounds from the whole plant by Venkataiah et al. (2003). Chhabra et al. (1999) discovered three ambrosanolides from the chloroform extract of this weed.

## Health hazards to humans and livestock

This weed is known to cause many health hazards which have now reached epidemic proportions. Agriculturists are concerned about *P. hysterophorus* affecting food and fodder crops, since the pollen and dust of this weed elicit allergic contact dermatitis in humans (Gunaseelan 1987; Morin et al. 2009). Dermatitis is a T cell-mediated immune injury and the disease manifests as itchy erythematous papules and papulovesicular lesions on exposed areas of the body (Akhtar et al. 2010). These effects have been related to cytotoxicity of the sesquiterpene lactone parthenin (Narasimban et al. 1984). Persons exposed to this plant for prolonged period manifest the symptoms of skin inflammation, eczema, asthma, allergic rhinitis, hay fever, black spots, burning and blisters around eyes. *Parthenium hysterophorus* also causes diarrhoea, severe papular erythematous eruptions, breathlessness and choking (Maishi et al. 1998). Exposure to *P. hysterophorus* pollens causes allergic bronchitis (Towers and Subba Rao 1992). Ramos et al. (2001) assessed the mutagenic potential of a crude extract of *P. hysterophorus* in the *Salmonella*/microsome (Ames) assay and the mouse bone marrow micronucleus test. However, it did not show genotoxic potential. Sharma et al. (2005) observed that the clinical pattern of Parthenium dermatitis progresses from airborne contact dermatitis to mixed pattern or chronic actinic dermatitis pattern. Eczema herpeticum is reported to complicate parthenium dermatitis. Sriramarao et al. (1993) worked on the use of murine polyclonal anti-idiotypic antibodies as surrogate allergens in the diagnosis of *P. hysterophorus* hypersensitivity. *Parthenium*-sensitive patients with rhinitis who had positive results on skin prick tests to *P. hysterophorus* pollen extracts responded with a positive skin reaction to *m*Ab-2. Akhtar et al. (2010) studied the involvement of T_H_ type cytokines in Parthenium dermatitis.

Exposure to *P. hysterophorus* also causes systemic toxicity in livestock (Gunaseelan 1987). Alopecia, loss of skin pigmentation, dermatitis and diarrhoea has been reported in animals feeding on *P. hysterophorus*. Degenerative changes in both the liver and kidneys and inhibition of liver dehydrogenases have been reported in buffalo and sheep (Rajkumar et al. 1988). The milk and meat quality of cattle, buffalo and sheep deteriorate on consumption of this weed (Lakshmi and Srinivas 2007). Significant reduction in rat WBC count after oral treatment of Parthenium extract signifies its immune system weakening ability (Yadav et al. 2010).

## Reducing agricultural and pasture productivity

Singh et al. (2003) explored the allelopathic properties of unburnt (UR) and burnt (BR) residues of *P. hysterophorus* on the growth of winter crops, radish and chickpeas. The extract prepared from both UR and BR was toxic to the seedling length and dry weight of the test crops. BR extract was more toxic due to its highly alkaline nature. Growth studies conducted in soil amended with UR and BR extracts revealed phytotoxic effects towards test crops, UR being more active than BR unlike crude extracts. These effects were attributed to the presence of phenolics (Singh et al. 2003). Parthenin leaching as root exudate plays a pivotal role in allelopathic interference with surrounding plants (Belz et al. 2007). Parthenin has also been reported as a germination and radicle growth inhibitor in a variety of dicot and monocot plants and it enters the soil through the decomposing leaf litter (Gunaseelan 1998). Burning of *P. hysterophorus* in fields reduced germination, biomass growth, plumule and radicle length of *Phaseolus mungo* (Kumar and Kumar 2010). Poor fruiting of leguminous crops and reduction in chlorophyll content of crop plants were observed in *P. hysterophorus*-infested fields (Lakshmi and Srinivas 2007). *Parthenium hysterophorus* played role as alternate host for crop pests functioning as an inoculum source. This weed has been reported to serve as a reservoir plant of scarab beetle, a pest of sunflower. *Parthenium hysterophorus* invasion causes changes in above-ground vegetation and below-ground soil nutrient contents, disturbing the entire grassland ecosystem in Nepal as reported by Timsina et al. (2010).

*Parthenium hysterophorus* is a serious invasive weed of pasture systems, reducing pasture productivity 90% (Evans 1997). It has become a major weed of grazing lands in central Queensland and New South Wales in Australia. It squeezes grasslands and pastures, reducing the fodder supply. Dhileepan (2007) observed dwindling effect of *P. hysterophorus* on grass biomass of grazing fields in Queensland, Australia.

## Biodiversity loss due to *P. hysterophorus*

The invasive capacity and alleolopathic properties have rendered *P. hysterophorus* with the potential to disrupt the natural ecosystems. Very sparse or sometimes no other vegetation can be seen in *P. hysterophorus*-dominated areas. It has been reported to be causing a total habitat change in native Australian grasslands, open woodlands, river banks and flood plains (Lakshmi and Srinivas 2007). These weeds rapidly invade new surroundings often replace the indigenous species and pose a serious threat to biodiversity in India. Akter and Zuberi (2009) conducted an extensive survey on invasive alien species (IAS) and their impact on different land use types viz. road side, low land, fallow land, homestead and railway track in Bangladesh. Among others, *P. hysterophorus* exhibited the ability to invade and adapt to new habitats, thereby reducing the number of indigenous plants. The more vigorous mode of reproduction and the possession of an array of secondary metabolites give the weed the status of invasive alien species.

## Disposal and eradication of *P. hysterophorus* weeds

*Parthenium hysterophorus* has multiple harmful aspects and no particular use. Its eradication is a major challenge to government, primarily because of its epidemic proliferation and strong reproductive potential, apart from its wide ecological range. Several physical and chemical methods used in the past to eliminate this weed have proved ineffective, expensive and not eco-friendly. The biomass of this plant is not put to any use and disposed along the roadsides, agricultural fields and railway tracks after uprooting. Further, these weeds are burnt in order to prevent various ailments induced by its toxic sesquiterpene lactone. However, burning of *P. hysterophorus* residues is not a recommended practice as it deteriorates the soil quality by rendering it more alkaline and deficient in organic matter (Singh et al. 2003). Tamado and Milberg (2004) conducted experiments to compare the effect of hand hoeing and applying herbicide (2,4-D) on growth of this weed and its effect on yield of sorghum in small holder farming systems in Ethiopia. Hoeing proved to be more efficient than the use of chemical herbicide.

## Biological control of *P. hysterophorus* weeds

**Fig. 3:**
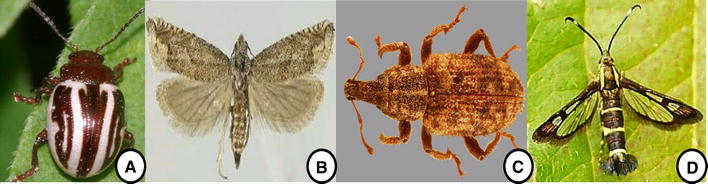
Biocontrol agents of *P. hysterophorus* (**a**) *Zygogramma bicolorata* (**b**) *Epiblema strenuana* (**c**) *Listronotus setosipennis* and (**d**) *Carmenta ithacae*

Dhileepan (2003a, b) studied the effectiveness of leaf-feeding beetle *Zygogramma bicolorata* (Fig. 3), stem-galling moth *Epiblema strenuana* and stem-boring weevil *Listronotus setosipennis* introduced against *P. hysterophorus* in Australia. The moth *Carmenta ithacae* and leaf-rust *Puccinia melampodi* were released to eliminate this weed, but little success has been attained in this regard as the weed has great regenerative potential and moreover the insect consumes only the foliage of the weed which stimulates further leafy proliferation (Dhileepan and Strathie 2009). The flowers and seeds, which are the main source of its dissemination, remain unaffected.

Eucalyptus, a native of Australia, is a known allelopathic tree that exerts its toxicity through leachates and volatile terpenes on adjoining vegetation/agricultural crops. The volatile terpenes present in leaves of eucalyptus emanate in the form of vapours into the surroundings. The vapours get adsorbed to soil, curbing the seed germination and reducing the chlorophyll content as well as cellular respiration. The oil vapours increase water loss leading to wilting. Eucalyptus oils may be used as natural herbicides for the biocontrol of *P. hysterophorus* owing to its allelochemicals (Kohli et al. 1998). *Cassia sericea* has the ability to overgrow *P. hysterophorus* in North-East India. Also marigold (*Tagetes erecta*) is reported to suppress *P. hysterophorus* growth in field trials (Lakshmi and Srinivas 2007).The control agents for the weed *P. hysterophorus* are listed in Tables 1, 2.

**Table 1:** Harmful effects of *P. hysterophorus*

Harmful effects	Reference
Reducing pasture productivity	Evans (1997)
Allelopathy, destroying agroecosystems	Singh et al. (2003)
Allergenic respiratory reactions and allergic contact	Gunaseelan (1987)
Dermatitis in humans and livestock	Morin et al. (2009)
Black spots, blisters around eyes, burning, rings and blisters over skin, asthma diarrhoea, severe papular erythematous eruptions, breathlessness and choking	Maishi et al. (1998)
	Lakshmi and Srinivas (2007) Akter and Zuberi (2009)
Biodiversity loss

**Table 2:** Biological control of *Parthenium hysterophorus*

Biocontrol agent of the weed	Reference
Leaf-feeding beetle *Zygogramma bicolorata*	Dhileepan (2003a, b)
Stem-galling moth *Epiblema strenuana*	
Stem-boring weevil *Listronotus setosipennis*	
Moth *Carmenta ithacae*	Dhileepan and Strathie (2009)
Leaf-rust *Puccinia melampodi*	
Fungus *Puccinia abrupta* var. *partheniicola* urediniospores	Fauzi et al. (1999)
Fungus *Alternaria* spp., *Fusarium* spp., *Rhizoctonia solani*,*Colletotrichum capsici*	Lakshmi and Srinivas (2007)
*Cassia sericea* and *Tagetes erecta*	Lakshmi and Srinivas (2007)
Eucalyptus oils	Kohli et al. (1998)
Flumioxazin, 2,4-D	Grichar (2006)

## Exploring future scopes of *P. hysterophorus*

Pink Morning Glory (*Ipomoea carnea*) and Spanish Flag *Lantana camara* are crucial examples regarding management of *P. hysterophorus*. *Ipomoea carnea* was introduced in India as green manure crop but later it posed a problem. But now, this weed has been discovered to possess many uses.

Ganesh et al. (2008) produced methane-rich gas from solid-feed anaerobic digestion of *I. carnea.* Patel et al. (2010) reported that a novel chitinase enzyme with potential use in agriculture, industry, environmental protection and chito-oligosaccharide production can be purified from the latex of *I. carnea*. Similarly, *L. camara* which was once brought to India as an ornamental plant encroaches on agricultural land and reduces the carrying capacity of pastures. Two novel triterpenoids have been isolated from the roots of *L. camara* L. Its leaves have yielded an essential oil which is rich in sesquiterpenes, and a hepatoprotective compound oleanolic acid is isolated from the roots (Misra and Laatsch 2000). Methanolic extract of *L. camara* leaves exhibit antiulcerogenic activity on gastric and duodenal ulcers in experimental rats (Sathish et al. 2011). It contains high amount of holocellulose and can serve as a low-cost feedstock for bioethanol production (Kuhad et al. 2010). The same strategy can be adopted for dealing with *P. hysterophorus*. Some future prospects envisioned for *P. hysterophorus* are presented in Table 3.

**Table 3:** Possible utilization of *P. hysterophorus*

Uses	Reference
Removal of heavy metals from environment to sequester Cd(II) ions from soil	Ajmal et al. (2006)
Sequestration of Ni(II) from aqueous solution onto activated carbon	Lata et al. (2008)
Eradication of salvinia and water hyacinth from water bodies	Pandey (1994)
Carbonized parthenium can be used for removal of dyes, heavy metals, nitrates and phenols	Rajeshwari and Subburam (2002)
PAC has excellent cresol adsorptive characteristic	Singh et al. (2008)
Additive with cattle manure in biogas production	Gunaseelan (1987)
Ovicidal, anti-fleedant, and nematocidal activity	Datta and Saxena (2001)
*P. hysterophorus* can be used as low-cost substrate for xylanase production	Dwivedi et al. (2009)
As compost; green manure for maize and mungbean production	Kishor et al. (2010); Javaid (2008)
Silver nanoparticles formation for biomedical uses	Parashar et al. (2009)
Parthenin exhibits significant anticancer property	Das et al. (2007)
Folk remedy against skin diseases, ulcerated sores, facial neuralgia, fever and anaemia	Venkataiah et al. (2003)
As analgesic in muscular rheumatism and vermifuge to eliminate helminths	Maishi et al. (1998)
Treat inflammation, eczema, skin rashes, herpes, rheumatic pain, cold heart trouble and as a remedy for female ailments	Maishi et al. (1998)
Treat fever, diarrhoea, neurologic disorders, urinary infections, dysentery, malaria and as emmenagogue	Surib-Fakim et al. (1996)
Flea-repellent for ridding dogs	Maishi et al. (1998)
Animal feed due to high potash, oxalic acids and protein	Mane et al. (1986)

## Health benefits of *P. hysterophorus*

The decoction of *P. hysterophorus* has been used in traditional medicine to treat fever, diarrhoea, neurologic disorders, urinary tract infections, dysentery, malaria and as emmenagogue (Surib-Fakim et al. 1996). Ethnobotanically, it is used by some tribes as remedy for inflammation, eczema, skin rashes, herpes, rheumatic pain, cold, heart trouble and gynaecological ailments. *Parthenium hysterophorus* has been found to be pharmacologically active as analgesic in muscular rheumatism, therapeutic for neuralgia and as vermifuge (Maishi et al. 1998). This weed is also reported as promising remedy against hepatic amoebiasis. Parthenin, the major constituent of the plant, exhibits significant medicinal attributes including anticancer property (Venkataiah et al. 2003). The methanol extract of the flowers showed significant antitumour activity and parthenin exhibited cytotoxic properties against T cell leukaemia, HL-60 and Hela cancer cell lines (Das et al. 2007). Previously, Ramos et al. (2002) had established the antitumour potential of *P. hysterophorus* extracts in vitro and in vivo with positive results in terms of tumour size reduction and overall survival of cell lines. Aqueous extract of *P. hysterophorus* has hypoglycaemic activity against alloxan-induced diabetic rats (Patel et al. 2008). So, flower extract of this weed can be used for developing drug for diabetes mellitus.

Parashar et al. (2009) reported the synthesis of silver nanoparticles by reducing silver ions present in the aqueous solution of silver nitrate complex using the extract of *P. hysterophorus.* This discovery can promote this noxious plant into a valuable weed for nanotechnology-based industries in future. Applications of such eco-friendly nanoparticles in bactericidal, wound healing and other medical and electronic applications makes this method potentially exciting for the large-scale synthesis of other nanomaterials.

## Role of *P. hysterophorus* in enhancement of crop productivity

Allelopathy can be used to increase crop production at minimal expenses and to diminish the current reliance on synthetic agrochemicals that degrade the environmental quality. The allelochemicals can be exploited as herbicides, insecticides, nematicides, fungicides and growth regulator. Pesticidal potential has been established in terms of ovicidal and anti-fleedant effects (Datta and Saxena 2001). The allelochemicals also provide defence against herbivorous predators.

Kishor et al. (2010) prepared compost of *P. hysterophorus* in 14 weeks and assessed its manure value. Compost from this weed on application in soil enhanced its moisture level more than nitrogen, phosphorus and potassium (NPK) alone. Anaerobic digestion of parthenium dried solids biodegrades the plant growth and conserves the NPK content. This can be applied as organic manure (Gunaseelan 1998). Javaid (2008) used *P. hysterophorus* weed as green manure for maize and mung bean production. The highest root and shoot biomass in maize was obtained in 3% green manure treatment, which was significantly greater than that obtained in the control and equivalent to that obtained in the NPK fertilizer treatments.

The effect of *P. hysterophorus* green manure and EM (effective microorganisms), a biofertilizer, on wheat (*Triticum aestivum* L.) cultivation was studied. Highest root biomass was recorded in 3% green manure-amended treatment. Spike length, number of grains per spike and grain yield gradually increased by increasing the quantity of green manure. There was 43–253% increase in grain yield over control due to various green manure treatments as compared with 96% increase due to NPK fertilizers over control (Javaid and Shah 2010). *Parthenium hysterophorus* being rich in N, P, K, Ca, Mg and chlorophyll content is ideally suited for composting. Ordinary *P. hysterophorus* compost cannot sufficiently reduce the allelopathic effects of high levels of parthenin and phenolics, which impede the early growth, development and dry matter yield of both monocot and dicot plants. For maximum exploitation of the nutrient contents of *P. hysterophorus*, without incurring the ill effects of phenolics, millipede *Harphaphe haydeniana*-mediated novel composting procedure was tried. This milli-compost (MC) was more effective than ordinary parthenium compost (OPC) (Apurva et al. 2010). So, if tapped properly, this weed can contribute to agronomic processes.

## Bioremediation of heavy metals and dyes by *P. hysterophorus*

Environmental pollution with heavy metals has become a global phenomenon. Nickel (II) is present in the effluents of silver refineries, electroplating, zinc base casting and storage battery industries. At higher concentrations, nickel causes cancer of lungs, nose and bone. Cost-effective alternative technologies or absorbents are needed for the treatment of metal-contaminted wastewaters especially in developing countries like India. Lata et al. (2008) studied the adsorption capacity of *P. hysterophorus* for the removal of nickel from aqueous solution by varying parameters such as agitation time, Ni(II) concentration, adsorbent dose and pH. The dried biomass of *P. hysterophorus* is used for carbon preparation by mixing it with concentrated sulphuric acid (1:1.5 w/v ratio) and keeping it at 120°C for 24 h, followed by washing and drying. This sulphuric acid-treated carbonized Parthenium (SWC) could be an effective, easily available and low-cost adsorbent for the removal of Ni(II) from dilute aqueous solution.

Cadmium (Cd) is widely used in electroplating, plastic manufacturing, metallurgical processes and industries of pigments and Cd/Ni batteries. However, it is extremely toxic even in low dosages and responsible for causing renal disorder, high blood pressure, bone deformity and destruction of RBCs. Because of bioaccumulation, Cd (II) is considered as a priority pollutant by the US Environmental Protection Agency. Ajmal et al. (2006) studied the efficiency of dried powder of *P. hysterophorus* as an adsorbent for removing Cd(II) from waste water. Batch process was employed for adsorption of Cd(II) ions by dried and crushed mass of *P. hysterophorus*. Atomic absorption spectrophotometry (AAS) of the filtrate showed that *P. hysterophorus* is an effective adsorbent over a wide range of initial Cd(II) concentration. The maximum adsorption of Cd(II) ions in the pH range 3–4 was 99.7%. The desorption studies showed 82% recovery of Cd(II) from the adsorbent, when 0.1 M HCl solution was used as effluent.

Cresol, a phenol derivative, is found in effluents of petrochemical, oil and metal refineries, chemical and glass fibre manufacturing, ceramic and steel plants, phenolic resin manufacturing industries, etc. This toxic effluent is known to cause stomach tumours, corrode the eyes, skin and respiratory tracts and affect the central nervous system, cardiovascular system, lungs, kidney and liver, even leading to unconsciousness and death. Activated carbon prepared from *P. hysterophorous* by chemical activation using concentrated H_2_SO_4_ is an effective adsorbent material. In order to test the adsorbent efficacy of parthenium-based activated carbon (PAC), it is compared with commercial grade activated carbon (AC). PAC is found to be as good as AC for removal of p-cresol up to a concentration of 500 mg/l in aqueous solution. AC is an expensive activated carbon and so regeneration is essential. In contrast to this, PAC is inexpensive, easily available and does not need regeneration and thus promises sustainable utilization in p-cresol removal from industrial wastewater (Singh et al. 2008).

The discharge of coloured waste into streams affects their aesthetic nature, reduces photosynthesis and renders aquatic bodies toxic due to the metals and chlorides in it. Adsorbents prepared from *P. hysterophorus* are tested to remove methylene blue from an aqueous solution in a batch reactor. Dye adsorption capacity of sulphuric acid-treated parthenium (SWC) and phosphoric acid-treated parthenium (PWC) is compared with that of commercially available activated carbon (AC). Maximum dye is sequestered by AC; however, PWC and SWC also showed significant results and can be considered as potential adsorbents for methylene blue removal from dilute aqueous solutions (Lata et al. 2007). Going by these promising findings, this weed can be exploited for industrial pollution control.

## Eradication of weeds by *P. hysterophorus*

Salvinia (*Salvinia molesta* Mitchell), water lettuce (*Pistia stratiotes*) and water hyacinth (*Eichhornia crassipes*) choke off water bodies suffocating aquatic creatures. Pandey (1994) studied the effect of dry *P. hysterophorus* L. leaf powder on these menacing weeds. The treatment caused wilting and desiccation of above-water parts of these floating plants. With the increasing concentration of *P. hysterophorus* extracts, the seed germination and growth of lovegrass (*Eragrostis)* decreased significantly (Tefera 2002).

## *P. hysterophorus* as substrate for enzyme production

Xylanases are hydrolytic enzymes that cleave xylans. The end products of xylan degradation have industrial applications for biofuel, artificial sweetener, animal feed production, baking and textile industry, clarification of fruit juices and coffee extraction. Besides, there has been an increasing interest in using xylanases for ecofriendly bleaching of pulp in paper industries. The potential of *P. hysterophorus* as low-cost raw material for xylanase production was studied by Dwivedi et al. (2009). They investigated xylanase production from a mutant of *Penicillium oxalicum* in submerged fermentation. Considerably higher level of the enzyme production in medium containing *P. hysterophorus* confirms the feasibility of using this cheap resource as an alternative carbon source to save costs of the enzyme production process (Dwivedi et al. 2009).

## *P. hysterophorus* as additive with cattle manure in biogas production

In the wake of oil crisis, energy generation from biowastes by anaerobic digestion has attracted immense attention. Energy crops are likely to be future sources of digester feed stocks for methane generation. *Parthenium hysterophorus* was mixed with cattle manure at a 10% level and allowed to digest anaerobically at room temperature in 3-l batch digesters. The chemical changes during the course of digestion and the effect of digested slurry (inoculum) on biogas production were investigated and significant increase in methane content was achieved. The methane content of the gas varied between 60 and 70% (Gunaseelan 1987). *Parthenium hysterophorus* should be seriously considered as a substrate for the production of biogas in India via anaerobic digestion, considering the abundance of this weed and large quantity of livestock.

## *P. hysterophorus* for welfare of livestock

*Parthenium hysterophorus* can be used as a flea-repellent for dogs (Maishi et al. 1998). This weed is a valuable source of potash, oxalic acids and high-quality protein (HQP) which can be used in animal feed (Mane et al. 1986).

## Discussion

Mechanical, chemical and biological control strategies have been proved futile individually to curb proliferation of *P. hysterophorus*. So, integrated approaches are warranted to restrict the invasion of this weed*.* To address this problem, public awareness has to be developed and participatory approach to control the invasive weeds should be adopted.

There is the need to encourage the research on the utilization potential of this weed and to evaluate its efficacy on field trials. The target of “control through utilization” can be achieved through joint efforts of researchers, farmers, governmental and non-governmental agencies. The discovery of the uses of this weed also could pave the way for indirect eradication of the weed. At present, although *P. hysterophorus* is considered a weed, its new uses are coming to the forefront. Nanomedicine, biopecticide, green manure potential, agent for bioremediation of toxic metals and dyes, herbicide, cheap substrate for enzyme production and source of biogas are some of the recently discovered implications of *P. hysterophorus*.

This weed is available in four continents in abundance. Their industrial processing costs are low and devoid of any environmental hazards. The increased utilization of *P. hysterophorus* biomass as energy source and raw materials is necessary in the long term, as fossil fuels are limited. Similarly, its use as manure and pesticide can be appreciated in the wake of the problems posed by chemicals. Isolation and chemical investigation of the compounds in *P. hysterophorus* are required to decipher their properties and predict their applications. In this regard, it is touted to become a boon for the human beings, animals and crops in near future.
